# Efficacy and Safety of Nutrient Supplements for Glycaemic Control and Insulin Resistance in Type 2 Diabetes: An Umbrella Review and Hierarchical Evidence Synthesis

**DOI:** 10.3390/nu14112295

**Published:** 2022-05-30

**Authors:** Charmie Fong, Simon Alesi, Aya Mousa, Lisa J. Moran, Gary Deed, Suzanne Grant, Kriscia Tapia, Carolyn Ee

**Affiliations:** 1 School of Medicine, Western Sydney University, Locked Bag 1797, Penrith, NSW 2751, Australia; 19431563@student.westernsydney.edu.au; 2 Monash Centre for Health Research and Implementation (MCHRI), School of Public Health and Preventive Medicine, Monash University, Level 1/43-51 Kanooka Grove, Clayton, VIC 3168, Australia; simon.alesi1@monash.edu (S.A.); aya.mousa@monash.edu (A.M.); lisa.moran@monash.edu (L.J.M.); 3 School of Public Health and Preventive Medicine, Monash University, 553 St Kilda Road, Melbourne, VIC 3004, Australia; drgarydeed@outlook.com; 4 NICM Health Research Institute, Western Sydney University, Locked Bag 1797, Penrith, NSW 2751, Australia; s.grant@westernsydney.edu.au (S.G.); kriscia.tapia@sydney.edu.au (K.T.); 5 L7/D18 Susan Wakil Health Building, Faculty of Medicine and Health, School of Health Sciences, University of Sydney, Western Avenue, Camperdown, NSW 2006, Australia

**Keywords:** type 2 diabetes, glycaemic control, insulin resistance, nutrients, umbrella review

## Abstract

Background: Nutrient supplements are widely used for type 2 diabetes (T2D), yet evidence-based guidance for clinicians is lacking. Methods: We searched the four electronic databases from November 2015–December 2021. The most recent, most comprehensive, high-ranked systematic reviews, meta-analyses, and/or umbrella reviews of randomised controlled trials in adults with T2D were included. Data were extracted on study characteristics, aggregate outcome measures per group (glycaemic control, measures of insulin sensitivity and secretion), adverse events, and Grading of Recommendations Assessment, Development, and Evaluation (GRADE) assessments. Quality was assessed using A Measurement Tool to Assess Systematic Reviews Version 2.0 (AMSTAR 2). Results: Twelve meta-analyses and one umbrella review were included. There was very low certainty evidence that chromium, Vitamin C, and omega-3 polyunsaturated fatty acids (Ω-3 PUFAs) were superior to placebo for the primary outcome of glycated hemoglobin (HbA1c) (Mean Difference/MD −0.54, −0.54 and ES −0.27, respectively). Probiotics were superior to placebo for HbA1c (Weighted Mean Difference/WMD −0.43%). There was very low certainty evidence that Vitamin D was superior to placebo for lowering HbA1c in trials of <6 months (MD −0.17%). Magnesium, zinc, Vitamin C, probiotics, and polyphenols were superior to placebo for FBG. Vitamin D was superior to placebo for insulin resistance. Data on safety was limited. Conclusions: Future research should identify who may benefit from nutrient supplementation, safety, and optimal regimens and formulations.

## 1. Introduction

In 2019, an estimated 463 million people (9.3% of the global population) were living with diabetes, with type 2 diabetes (T2D) accounting for 90% [1]. This is predicted to rise to 10.2% (578 million) by 2030 and 10.9% (700 million) by 2045 [1]. Worldwide, diabetes was the ninth leading cause of death in 2019 [2], posing an immense burden on healthcare systems due to morbidity, including a two-fold risk for coronary artery disease and stroke [3].

Type 2 diabetes is characterised by β-cell dysfunction and progressive loss of β-cell insulin secretion, often against a background of insulin resistance [4]. The goals of treatment for type 2 diabetes are to prevent or delay complications and optimise quality of life [5]. Nutrition therapy, weight management, self-management, and physical activity are all recommended as cornerstones of diabetes management in order to achieve diabetes treatment goals [5]. Glycaemic control is fundamental to managing T2D. A HbA1c goal of <7% is recommended for many nonpregnant adults, although it is recommended that this target is individualised, taking into account individual needs and preferences, and issues that affect safety and adherence. HbA1c levels have strong predictive value for both macro and microvascular diabetes complications [6,7]. Insulin resistance is one of the key pathophysiological factors for developing T2D [8,9,10], and approaches to manage T2D include measures to improve insulin sensitivity, such as physical activity [11].

More than a quarter (26.2%) of adults with diabetes in the United States reported using some form of complementary medicine in 2012, including nutrient supplements [12]. Global prevalence may be even higher; a 2020 meta-analysis of complementary medicine use in adults with diabetes found a wide variation (predictive interval between 8–93%), with a pooled prevalence of 51% [13]. Use of nutrient supplements in the general population is also growing [14], with data collected in the United States and Australia indicating an increasing trend over time [15,16], while their use in Europe varies significantly between countries [17].

Despite the widespread use of nutrient supplements among people with T2D, there is currently a lack of clear guidance for clinicians regarding the use of nutrient supplements for diabetes management. The American Diabetes Associations’ Standards of Medical Care in Diabetes 2022 states that there is no clear evidence that dietary supplementation with vitamins or minerals can improve outcomes in people with diabetes who do not have underlying deficiencies, and they are not generally recommended for glycaemic control [11]. However, this recommendation is based on older literature published in 2008–2014 [18,19,20,21,22]. Nutrient supplements may indeed play a role in managing T2D, as people with T2D can be more prone to underlying micronutrient deficiencies; there are also biologically plausible mechanisms by which nutrient supplements may act to improve glycaemic control [23,24]. Last, to aid informed decision-making and patient-centred care (which is respectful and responsive to patient preferences, needs, and values) when patients enquire about use of nutrient supplements in T2D, a discussion of the most recent and best available evidence is needed.

We conducted an umbrella review with the aim of aggregating top-tier evidence from systematic reviews and meta-analyses (MA) of randomised controlled trials (RCTs) that examine the efficacy and safety of individual nutrient supplements for glycaemic control and insulin resistance in individuals with T2D.

## 2. Materials and Methods

An umbrella review, also referred to as a review of reviews or overview of systematic reviews, uses explicit and systematic methods to search for, identify, extract data from, and analyse the results of multiple related systematic reviews with the aim of providing a single synthesis of systematic review evidence. Currently, there are no published guidelines for reporting of umbrella reviews, although guidelines are currently under development (the Preferred Reporting Items for Overviews of Reviews/PRIOR guidelines) [25]. A protocol was developed a priori conforming to the PRISMA guidelines and informed by the work by Lunny, et al. [26] regarding managing overlapping and discordant data. Our protocol is registered on Open Science Framework (https://osf.io/e9cjp (accessed on 3 May 2022)).

### 2.1. Search Strategy

One reviewer (CF) conducted a systematic search using Cochrane Database of Systematic Reviews, PsycINFO, MEDLINE, and EMBASE from November 2015–November 2020, with the search updated in December 2021. We limited our search to the five years preceding the first search in order to capture only the most recent evidence. Studies were limited to SRs and MAs and English language only. Supplementary Table S1 shows the search strategy applied. Two reviewers (CF, CE) independently screened at least 10% of titles and abstracts for eligibility in duplicate, aiming to achieve 80% consistency, with the remainder of titles and abstracts screened by one reviewer (CF) and validated by a second reviewer (CE) who cross-checked all decisions for inclusion and exclusion. All full text articles were screened in duplicate (CF/CE). Disagreements were resolved by discussion.

### 2.2. Selection Criteria

Study design: Our inclusion criteria were umbrella reviews, meta-analyses, or systematic reviews (SR) of RCTs using nutrient supplementation as an adjunctive or stand-alone treatment for T2D. Due to the broad scope of our research question, it will not be feasible to gather all available data from every existing review of the interventions explored. Thus, we have developed a systematic approach towards data gathering, favouring the top-tiers of evidence synthesis. Using a hierarchical evidence synthesis method, we included the most recent umbrella reviews or systematic reviews/meta-analyses for each nutrient supplement, favouring the top tiers of evidence synthesis in the following order: (1) Umbrella reviews; (2) Network meta-analyses; (3) Meta-analyses of double-blind, placebo-controlled RCTs; (4) Meta-analyses of RCTs; (5) Systematic reviews of RCTs.

Managing overlapping reviews: If an umbrella review and an SR/MA addressed the same clinical question, we included the umbrella review. If the SR was more recent, we updated the results of the umbrella review with the newer SR. Where more than one similar review was identified with the potential for overlap, we retained the review with the most recent search end-date. Where there was uncertainty about the amount of overlap, we assessed primary study overlap by producing a citation matrix and calculating the Corrected Covered Area as per Pieper, et al. [27]. If there was very high overlap (>15%), we chose the most recent and then the most comprehensive review (i.e., the review that included the most RCTs).

Selection criteria according to the PICO (population, intervention, comparison, and outcome) framework are outlined below.

Population: We included studies on individuals with T2D. Studies were excluded if they focused on populations with prediabetes, metabolic syndrome, or overweight/obesity, without including any subgroup analyses for individuals with T2D.

Intervention: Nutrient supplements were defined as individual vitamins, minerals, pre/probiotics, fatty acids, or amino acids provided in supplement form. Studies on food products alone, fortified food interventions, diet composition, or dietary patterns and herbal supplements were excluded. We examined individual nutrient supplements in this review and not multi-nutrient formulations in an attempt to isolate the efficacy of individual nutrients prior to exploring the role of combinations of nutrients.

Comparison: There were no restrictions in terms of comparator groups.

Outcomes: Primary outcome measures were fasting blood glucose (FBG), glycated haemoglobin (HbA1c), and any adverse events (AEs). Secondary outcomes were measures of insulin sensitivity and secretion (Homeostatic Model Assessment of Insulin Resistance (HOMA-IR), Quantitative Insulin-Sensitivity Check Index (QUICKI), fasting insulin, or others).

### 2.3. Data Extraction

Two reviewers (SA and CF) independently extracted data using a predefined data extraction tool developed using Microsoft Excel. We extracted the number of trials and participants, dates of literature searches, sample details, intervention, and control details, weighted or standardised mean differences and confidence intervals for outcomes, adverse event reporting where available, risk of bias findings, and Grading of Recommendations Assessment, Development, and Evaluation (GRADE) assessments where available. As we did not intend to re-analyse data for specific subgroups or different summary measures, we summarised outcome data in a table, and no additional analyses were conducted [28].

### 2.4. Quality Assessment

Included studies were assessed using “A Measurement Tool to Assess Systematic Reviews” Version 2.0 (AMSTAR 2) [29]. From four nominated reviewers (CF, CE, SA, AM, LM), two independently applied the checklist to each study and any discrepancies were resolved via discussion. The number of critical and non-critical domains which were not adhered to were used to generate an overall rating of the article quality, with the confidence in the results of the review ranging from “critically low” to “high” as described by Shea, et al. [29]. The AMSTAR 2 critical domains are protocol registration before commencement of the review (item 2); adequacy of the literature search (item 4); justification for excluding individual studies (item 7); risk of bias from individual studies included in the review (item 9); appropriateness of meta-analytical methods (item 11); consideration of risk of bias when interpreting the results of the review (item 13); and assessment of presence and likely impact of publication bias (item 15). A rating of “critically low” represents more than one critical flaw with or without non-critical weaknesses; “low” represents one critical flaw with or without non-critical weaknesses; “moderate” represents more than one non-critical weakness but no critical flaws; and “high” represents one or no non-critical weakness.

### 2.5. Certainty of Evidence

The GRADE [30] tool was used to assess and report the certainty of evidence (i.e., confidence in the effect estimate) for each pre-defined, clinically important outcome of interest. If possible, these data were extracted directly as presented in the included reviews. Where GRADE assessments were not conducted by the systematic review authors, two reviewers (SA, CE, AM) conducted GRADE assessments independently using the information reported in the systematic review. Any disagreements were resolved by discussion. In some cases, particularly for reviews which focused on broader populations (e.g., individuals with overweight/obesity irrespective of T2D status), GRADE assessments were not possible since some reviews did not report individual RCT risk of bias in subgroups of people with T2D.

## 3. Results

### 3.1. Search Results

**Figure 1:**
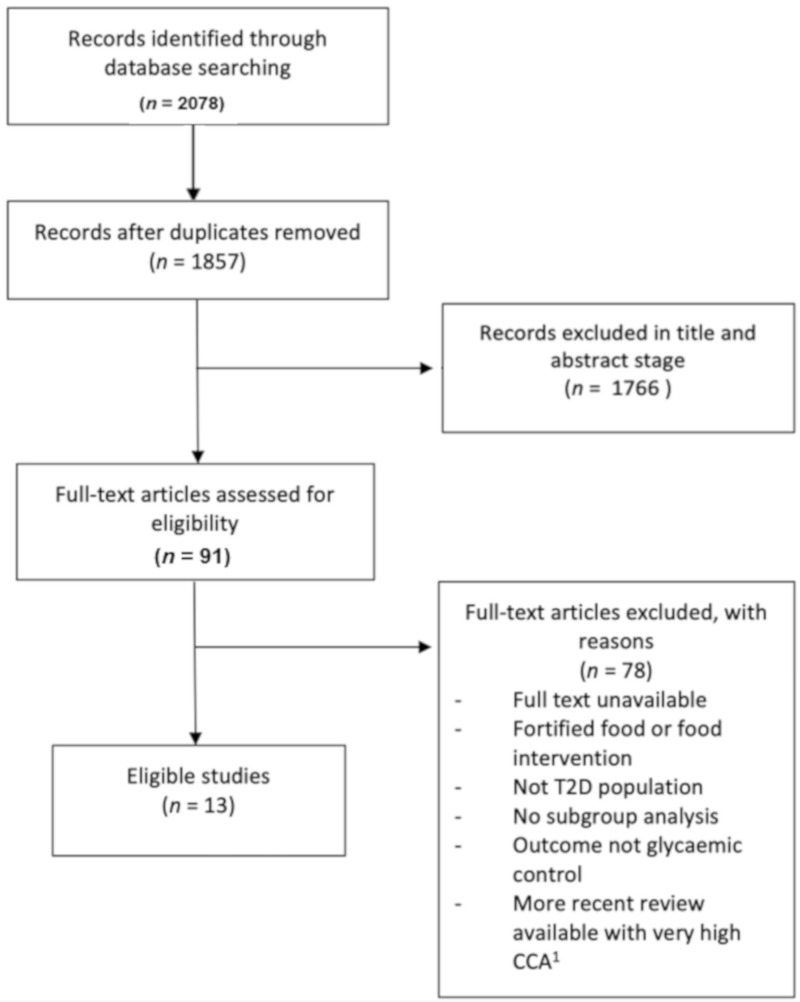
Flow chart of search results. ^1^ Corrected Covered Area: degree of overlap between reviews.

The search returned 2078 results, which reduced to 1857 after duplicates were removed. After title and abstract screening, 91 articles remained. After full text screening and removal of overlapping reviews, 13 remained eligible (Figure 1).

### 3.2. Study Characteristics

Of the 13 included reviews, one was an umbrella review [31], with the remainder being meta-analyses. Specifically, there were two meta-analyses on polyphenols [32,33], three on polyunsaturated fatty acids (Ω-3 PUFAs) [34,35,36], and one each for vitamin D [37], probiotics [38], magnesium [39], chromium [40], folate [41], and zinc [42]. We included one meta-analysis [43] and umbrella review [31] on vitamin C (Table 1). All meta-analyses only included randomised double-blind placebo-controlled trials. All included reviews were conducted between 2017 and 2021, with six of the fourteen (43%) published between 2020 and 2021 [32,33,35,38,40,43]. Seven reviews included only people with T2D [32,35,36,37,40,43,44], while two included adults with no restriction on health status or conditions [31,41], and five reviews included people with T2D, type 1 diabetes, prediabetes, gestational diabetes, obesity, or otherwise at high risk of T2D [33,34,38,39,42]. Unless otherwise indicated, we report subgroup analyses for people with T2D in Table 1.

All reviews analysed data on both FBG and HbA1c, except for Raimundo, et al. [33] who did not conduct any subgroup analysis on HbA1c for people with T2D. All but three reviews [34,38,40] analysed data on insulin resistance.

### 3.3. Quality Assessment Using AMSTAR 2

There was only one review with no critical weaknesses, and this was Jeyaraman, et al. [32] on resvaratrol. The remaining reviews all had one or more critical weakness, with almost half (5 of the 12 reviews: 42%) having three or more critical weaknesses. The umbrella review by Ashor, et al. [31] for vitamin C assessed the quality of their included systematic reviews using the Overview Quality Assessment Questionnaire (OQAQ) which found one review to be of moderate quality, while the rest were of high methodological quality. See Table 2 for a summary of the AMSTAR 2 assessments. 

### 3.4. Efficacy of Nutrient Supplementation in Glycaemic Control

There was very low certainty evidence that chromium, Vitamin C, and Ω-3 PUFAs were superior to placebo for the primary outcome of HbA1c, although Ω-3 PUFAs in the form of fish oil supplements [35] or alpha-lipoic acid [34] were not found to be efficacious. Probiotics were also reported to be superior to placebo for HbA1c [38], but a GRADE assessment was not possible due to insufficient reporting of individual RCT risk of bias by the authors. There was very low certainty evidence that Vitamin D was superior to placebo for lowering HbA1c based on a subgroup analysis of short-term (<6 month duration of intervention) trials [37]. Magnesium, zinc, Vitamin C, probiotics, and polyphenols were superior to placebo for FBG. There was no evidence to support the use of folate for glycaemic control in T2D [41]. All but one meta-analysis (which reviewed vitamin D) [37] reported no differences between groups for measures of insulin resistance. A summary of the results for each nutrient is provided below.

#### 3.4.1. Vitamin C

A 2021 meta-analysis by Mason, et al. [43] found that vitamin C could reduce FBG (MD −0.74 mmol/L, CI −1.17 to −0.31), HbA1c (MD −0.54%, CI 0.90 to −0.17) and post-prandial glucose (MD −0.95 mmol/L, CI −1.83 to −0.06). However, there was significant heterogeneity for all glycaemic outcomes and very low certainty of the evidence upon GRADE assessment conducted by the review authors. Vitamin C appeared to be more effective for glycaemic control when given for longer than 12 weeks, and in individuals with higher HbA1c at baseline. For each 1% increase in baseline HbA1c, vitamin C reduced HbA1c by −0.47%. There was no modifying effect from baseline Vitamin C levels. However, an increase in dose appeared to result in an increase in FBG; with each 100 mg/day increase in dose, vitamin C increased FBG by 0.09 mmol/L. An earlier 2019 umbrella review by Ashor, et al. [31] similarly reported a reduction in FBG with vitamin C (based on two meta-analyses), but no difference was reported for HbA1c (based on one meta-analysis). For the analysis on HbA1c, there was overlap on six trials between the umbrella review and newer meta-analysis. The newer meta-analysis by Mason, et al. included ten additional trials, of which eight had been published after the umbrella review was conducted.

Although one of the older systematic reviews included in Ashor’s umbrella review found a significant decrease in fasting insulin [45], the newer meta-analysis by Mason did not find any differences between vitamin C and placebo for fasting insulin or HOMA-IR. We were unable to determine the degree of overlap between the umbrella review and the newer meta-analysis for the outcomes of insulin resistance as the included trials in the umbrella review were not cited. However, in the meta-analysis of fasting insulin levels by Mason, et al., five out of the nine included trials were published after the last search date of the most recent systematic review in Ashor’s umbrella review.

#### 3.4.2. Chromium

There was very low certainty evidence that chromium lowered HbA1c (MD −0.54%, CI −0.98 to −0.09) but not FBG when compared with placebo based on a 2021 meta-analysis. Subgroup analysis found that only short-term intervention (<12 weeks) showed a statistically significant improvement in HbA1c, while dosage had no impact [40].

#### 3.4.3. Probiotics

In a meta-analysis by Cao, et al. of 17 studies, probiotic supplementation was associated with statistically significant, though modest, reductions in FBG and HbA1c in T2D compared with placebo (WMD −9.48 mg/dL and −0.43%, respectively). Of the included trials, ten used probiotics as the intervention, while seven used symbiotics (a combination of pre and probiotics). The most commonly used species across trials was from the *Lactobacillus* genus (all trials) and the *Bifidobacterium* genera (nine trials) [38], with commonly used species being *L. acidophilus, L. casei, L. reuteri*, *B. bifidum*, and *B. longum.* GRADE assessment was not possible due to insufficient reporting of individual RCT risk of bias; however, most of the included trials (which included trials in people with prediabetes or gestational diabetes mellitus) were at an unclear or high risk of bias when assessed by the review authors, and publication bias could not be ruled out.

#### 3.4.4. Zinc

Wang, et al. [42] conducted a meta-analysis on zinc supplementation on glycaemic control for diabetes prevention and management. In a subgroup analysis of people with T2D, there was very low certainty evidence that zinc supplementation reduced FBG (WMD −20.34 mg/dL, CI −29.04 to −11.64) but not HbA1c or measures of insulin resistance (HOMA-IR, fasting insulin) when compared with placebo.

#### 3.4.5. Magnesium

A 2017 meta-analysis [39] reported that magnesium supplementation reduced FBG (WMD −6.253 mg/dL, CI −6.253 to −1.904) but not HbA1c or fasting insulin in the subgroup of people with T2D, compared with placebo. GRADE assessment was not possible due to insufficient reporting of individual RCT risk of bias for subgroups of people with T2D.

#### 3.4.6. Polyphenols

A 2020 Cochrane review [32] concluded that there was insufficient evidence to determine the safety and efficacy of resveratrol supplementation for treatment of T2D, with no statistically significant difference between resveratrol and placebo for HbA1c, FBG, or insulin resistance (three trials, *n* = 50). Raimundo, et al. [33] examined several types of polyphenols (resveratrol, isoflavones, flavanols, and polyphenol mixture) in people with prediabetes or T2D. In a subgroup analysis of people with T2D, polyphenols lowered FBG compared to placebo (MD −5.86 mg/dL, CI −11.34 to −0.39), with a greater effect seen in those taking anti-diabetic medication (MD −10.17 mg/dL). A subgroup analysis for individual types of polyphenols was not conducted in people with T2D. A GRADE assessment was not possible due to insufficient reporting of individual RCT risk of bias for subgroups with T2D. There was no evidence that polyphenols improved insulin resistance. There was overlap of one RCT [48] between the meta-analyses conducted by Raimundo [33] and Jeyaraman [32].

#### 3.4.7. Ω-3 PUFAs

There was very low certainty evidence that Ω-3 PUFAs lowered HbA1c compared to placebo based on a 2018 meta-analysis [36] (ES −0.27, CI −0.48 to −0.06), with no effect seen for FBG or insulin resistance. However, “leave one out” sensitivity analysis revealed that the removal of two RCTs rendered the results non-significant. The two RCTs referred to in this analysis were not specified. There was very low-quality evidence from two additional meta-analyses that Ω-3 PUFAs administered in the form of fish oils [35] and alpha-lipoic acid [34] were not superior to placebo in improving HbA1c or FBG.

#### 3.4.8. Vitamin D

There was very low certainty evidence that vitamin D supplementation was not superior to placebo for FBG or HbA1c [37]. Further subgroup analysis found a small but statistically significant decrease in HbA1c (SMD −0.17%) in the short-term intervention subgroup (<6 months). Vitamin D lowered HOMA-IR (SMD −0.60, CI −0.79 to −0.42) and fasting insulin (SMD −0.49, CI −0.68 to −0.31) compared with placebo.

#### 3.4.9. Folate

There was very low certainty evidence that folate, given in the forms of folate or folic acid [41], was not superior to placebo for improving HbA1c, FBG, or HOMA-IR. There was no difference in outcomes in a subgroup analysis of baseline folate concentrations, however this analysis was conducted on the pooled sample which included trials in people without T2D.

#### 3.4.10. Safety of Nutrient Supplementation

Data on safety were limited. Only two reviews collected data on adverse events (AEs). Vitamin C appeared to be safe in people with T2D with no AEs reported across five RCTs, while two RCTs reported fewer AEs in the vitamin C group compared with placebo [49]. There was no difference between groups for renal and hepatic function tests. Similarly, no AEs were reported in three RCTs of resveratrol [32]. In their meta-analysis of vitamin D, Hu, et al. [37] stated that they extracted data on AEs, but the results for these were not reported in the review.

#### 3.4.11. Certainty of the Evidence

Two reviews reported GRADE assessments [32,49]. GRADE assessments were not possible for three reviews (on probiotics, magnesium, and polyphenols) due to insufficient reporting of individual trial risk of bias [33,38,39]. All GRADE assessments indicated very low certainty of the evidence and therefore very little confidence in the effect estimates (Table 1).

## 4. Discussion

To the best of our knowledge, this is the first umbrella review to comprehensively and critically evaluate top-tier evidence on nutrient supplements for T2D. Using a hierarchical evidence synthesis method, our aim was to facilitate informed clinical decision-making when patients with T2D are considering use of nutrient supplements for improving glycaemic control. Overall, there was very low-quality evidence that chromium, Vitamin C, Ω-3 PUFAs, and probiotics had a statistically significant impact on HbA1c compared with placebo. Evidence on the use of probiotics, zinc, vitamin D, polyphenols, and magnesium for improving fasting blood glucose appears promising, although certainty and quality of the evidence was generally very low. Vitamin D was the only nutrient that demonstrated superiority over placebo for insulin resistance.

Chromium reduced HbA1c by a mean of −0.54% compared with placebo but with no differences in FBG [40]. This is of clinical significance since the magnitude of effect is similar to most oral hypoglycaemic drugs, which reduce HbA1c levels by 0.5–1.25% [50]. Indeed, preclinical studies have demonstrated that chromium may have similar mechanisms of action to some oral hypoglycaemic drugs. For instance, chromium may increase glucose uptake in skeletal muscle via GLUT4 translocation [51] and an increase in mRNA levels for members of the insulin-signalling cascade such as insulin receptor substrate (IRS)-1 and 2, PI3-kinase, and protein kinase B as well as AMP-activated protein kinase (AMPK) [52]. These actions are similar to those proposed for the common anti-diabetic drug, metformin [53]. The effects of chromium did not appear to be dose-dependent (based on a 200 μg cut-off). On subgroup analysis, a shorter (<12 weeks) but not longer timeframe was statistically significant for intervention which may be related to factors including high heterogeneity within the included RCTs, particularly the wide variation in the forms, doses and durations of chromium studied. For these reasons, and others, the certainty of the evidence is very low. An earlier systematic review on chromium for T2D reported no differences in AEs between intervention and placebo groups [54], however the safety of long-term administration of chromium remains unclear as the maximum duration of administration in RCTs was 25 weeks [40].

Vitamin C may be safe and efficacious for glycaemic control in T2D (MD of −0.54% for HbA1c), particularly for individuals with higher baseline HbA1c with vitamin C administered for greater than 12 weeks. These findings are likely to be clinically significant [50]. However, given the majority of studies administered vitamin C for <6 months, there is limited data on the safety and efficacy of longer-term administration. The dose administered also varied considerably from 200 mg to 3 g daily, and higher doses may increase, rather than decrease, FBG. Additionally, certainty of findings for HbA1c are very low. It is unclear what the potential mechanism of action may be, although it is postulated that vitamin C’s antioxidant properties play a role in glycaemic control, with vitamin C administration significantly increasing insulin-mediated glucose disposal, ameliorating skeletal muscle oxidative stress, and reducing free radical activity [49,55].

Cao, et al. [38] reported a significant effect of probiotics on HbA1c (−0.43%) compared with placebo, as well as a modest reduction in FBG (WMD −9.48 mg/dL), although both these effects are unlikely to be clinically significant [50]. The main species were from the *Lactobacillus* and *Bifidobacterium* genera. The proposed mechanism of action of probiotics is centred on links between gut dysbiosis, systemic inflammation, and obesity. Metagenome-wide association studies have demonstrated that individuals with T2D have lower levels of butyrate-producing and higher levels of pathogenic species such as *Lactobacillus gasseri* and *Streptococcus mutans*, with an overall proinflammatory signature in the gut microbiota. Short-chain fatty acids (SCFA) such as butyrate activate G-protein coupled receptor (Gpr) 43 result in suppression of insulin signalling in adipose tissue and prevent fat accumulation, as well as enhancing insulin sensitivity via promotion of glucagon-like peptide-1 (GLP1) secretion in the gut [56]. Probiotics may influence glucose metabolism via modulation of the gut microbiome, thereby increasing SCFA secretion and subsequent production of GLP-1, a satiety signal often impaired in people with obesity [57]. GLP-1 is also an incretin hormone, stimulating insulin release in response to food intake (via regulation of ion channels) and inducing expansion of insulin-secreting β-cell mass [58]. Although Cao, et al. [38] did not report on AEs, another recent systematic review [59] on probiotics for T2D reported no serious AEs across 15 RCTs, while minor AEs such as abdominal cramping, dyspepsia, and diarrhoea in <5% of participants were reported in three RCTs. It is possible that use of multi-strain formulations may have a greater effect on glycaemic control, based on the pooled subgroup analyses from Cao’s meta-analysis (which combined people with T2D, prediabetes, and gestational diabetes mellitus). Multi-strain formulations have been postulated to be more efficacious due to providing a broader spectrum of efficacy, and potential additive or synergistic effects leading to benefits such as enhanced adhesion; however, their benefits over single-strain formulations remain unclear [60]. Confirmation of this awaits further trials with adequate control for dietary patterns.

Zinc supplementation was found to reduce FBG, but not HbA1c, compared with placebo. Zinc deficiency in individuals with T2D is common due to increased urinary excretion [61] and is associated with glucose intolerance and insulin resistance [62]. As an essential trace element, zinc is crucial for adequate function of over 300 enzymes playing a role in processes including DNA/RNA synthesis, cell division, and apoptosis [63]. Zinc is present in high amounts in β cells in the healthy pancreas, and an early seminal study reported a 75% decrease in pancreatic zinc content in people with T2D [64]. Early studies also demonstrated zinc is required to trigger insulin crystallisation, which increases the storage capacity of insulin-secreting vesicles [65]. Zinc potentiates the effects of insulin at the level of the insulin receptor [63] and also independently exerts insulin-like effects on skeletal muscle via induction of the phosphorylation of insulin receptors and protein-kinase B [66]. The insufficient timeframe of most of the included RCTs (*n* = 30 of the 36 trials were of ≤3 months duration [42]) may explain the discrepancies between FBG and HbA1c results, as changes in HbA1c would not be expected to be demonstrated in under 12 weeks.

Vitamin D was reported to be more efficacious than placebo for insulin resistance (HOMA-IR), and subgroup analyses on intervention duration reported a small but statistically significant reduction in HbA1c (−0.17%) with a duration of <6 months [37]. Observational studies have demonstrated an association between vitamin D deficiency and the onset and progression of T2D [67], and it has been suggested that vitamin D supplementation exerts a hypoglycaemic effect by stimulating pancreatic beta cell function [68]. Further, insulin secretion is a calcium-dependent process, with L-type calcium channels on beta-cells activated by vitamin D, which then promotes insulin signalling and release [69]. Similarly, current evidence has demonstrated the role of oxidative stress [70] and chronic low-grade inflammation [71] in the pathogenesis of diabetes. Thus, the use of anti-inflammatory and antioxidant supplements such as vitamin D [72] for glycaemic control in T2D has biological plausibility but its efficacy remains uncertain; it is also unknown what the optimal dose would be. Although Hu, et al. [73] did not report on safety, there have been no safety concerns hitherto with administration of vitamin D to individuals with T2D.

While this review has highlighted the potential efficacy of certain nutrient supplements for T2D management, the overall confidence in the evidence presented is critically low when assessed using AMSTAR 2 and there is very low certainty of evidence based on GRADE assessments. Many of the systematic reviews had several critical flaws potentially affecting their validity. The most common flaw (present in 11 reviews) was a lack of justification for excluding individual studies (Item 7), which may have led to biased results. Three reviews failed to use a satisfactory technique for assessing the risk of bias in individual studies (Item 9), and three reviews failed to register the protocol before commencement of the review (Item 2). Other critical domains which were not met in some systematic reviews included unclear principles on which meta-analyses were performed (Item 11), failure to demonstrate a comprehensive literature search strategy (Item 4), and inadequate assessment of publication bias (Item 15). Future systematic reviews and meta-analyses should include report data on excluded studies, use validated methods for assessing risk of bias, and use comprehensive search strategies. The certainty of evidence was downgraded for risk of bias, imprecision, inconsistency, and indirectness. Therefore, despite the promising findings for certain nutrient supplements, the certainty of these findings remains unclear. Future RCTs should minimise risk of bias by implementing adequate allocation concealment and blinding, use sample size calculations to ensure adequate statistical power, and administer the intervention for sufficient durations.

An important strength of this review is the inclusion of the most recent and highest ranked evidence. To our knowledge, there have been no previous umbrella reviews of this nature examining nutrient supplements for glycaemic control in T2D. We also conducted GRADE assessments wherever possible to assess the certainty of the evidence. With regard to limitations, we cannot determine comparative effectiveness from this umbrella review. A network meta-analysis may address this issue. Our hierarchical evidence synthesis approach means that not all systematic reviews were reported; however, our approach has the advantage of presenting only the most recent and most comprehensive evidence, therefore facilitating clinician access to the “best available evidence”. There was significant heterogeneity between included studies, with multiple variations in supplement formulations. Additionally, we did not examine the effect of combinations of nutrient supplements. There is a need for future research to explore this, given the common use of supplement preparations containing more than one micronutrient [16].

This review highlights the need for more rigorous RCTs and systematic reviews, with a focus on meeting the aforementioned critical components to reach moderate to high confidence in reported findings. This is of paramount importance if results are to be applied in clinical practice to improve outcomes and to ensure patient safety. Future reviews should aim to stratify the effects of these nutrient supplements across different demographics including by age, ethnicity, and comorbidity, as well as by dose, duration, and formulation, and to establish safety parameters and underlying mechanisms and interactions between commonly used supplements. Only two meta-analyses conducted subgroup analyses according to baseline vitamin/mineral concentrations [41,43]. Future research should also clearly identify if nutrient supplements have a greater benefit in people with identified nutrient deficiencies at baseline. Other sub-populations who would most benefit from supplementation should also be identified, such as people with poorer glycaemic control at baseline.

## 5. Conclusions

High-quality evidence supporting the use of nutrient supplements for improving glycaemic control or insulin resistance in T2D is lacking and currently insufficient to support their use in a clinical context. Future research should identify the most effective nutrient supplements or multi-nutrient formulations, their safety profiles and optimal doses and durations, and any subgroups of individuals who may benefit most from these supplements as adjuncts and/or alternatives to conventional treatments.

## Figures and Tables

**Table 1:** Characteristics and main findings of reviews included.

Author and Year (Review Type)	N of Participants (Trials) Inclusion Criteria	Intervention (Control Type)	Outcomes—Glycaemic Control	Outcomes—Insulin Resistance	Certainty of Evidence for the Primary Outcome: HbA1c (GRADE)
**VITAMIN C**					
Ashor, A., et al. 2019 [31] (UR) Inception–Feb 2018. Date of search for most recent MA (Ashor 2017) was February 2016.	N= 6409 (10 SR/MA; 3 on T2D) P = SRs and Mas of RCTs in adults of any health status; I = vitamin C administered alone; C = placebo; O = biomarkers of CVD risk (arterial stiffness, blood pressure, endothelial function, glycaemic control, and lipid profile).	Ashor, et al. 2017: duration of 28 to 120 days, dose administered varied between 500 and 2000 mg per day [45] Tabatabaei-Malazy, et al. 2014: duration 4 weeks to 9 years, dose between 200 to 1000 mg/day [46] Khodaeian, et al. 2015: duration 4 to 16 weeks, dose between 800–1000 mg/day [47]	↓FBG: SMD −20.59 (CI −40.77 to −0.4, 5 trials, *n* = NR) [46] ↓FBG: WMD −0.41 mmol/L (CI −0.78 to −0.04, 15 trials, *n* = 469) [45] HbA1c: NS (9 trials, *n* = NR) [45]	Insulin: NS except for ↓fasting insulin in Ashor, et al. 2017: WMD: −15.67 pmol/L (CI −31.61 to 0.27, 3 trials, *n* = NR) [45] HOMA-IR: NS (3 trials, *n* = 92) [47]	NA
Mason, S., et al. 2021 [43] (MA) Inception–September 2020	N = 1574 (28 trials) P = RCTs in people with T2D; I = vitamin C; C = placebo; O = HbA1c, FBG, PPG, FI, HOMA-IR, clamp insulin sensitivity, lipids, BP, oxidative stress markers 10 trials published after the Ashor umbrella review.	Oral vitamin C supplementation Dose range: 200 to 3000 mg daily Duration: 2 weeks to 1 year, majority of studies <6 months duration (*Placebo*)	↓FBG: MD −0.74 mmol/L (CI −1.17 to −0.31, 19 trials, *n* = 1305) ↓HbA1c: MD −0.54% (CI −0.90 to −0.17, 16 trials, *n* = 1133) ↓PPG: MD −0.95 mmol/L (CI −1.83 to −0.06, 4 trials, *n* = 235). No modifying effect from baseline Vitamin C concentration	Fasting insulin (9 trials, *n* = 436), HOMA-IR (5 trials, *n* = 263) and clamp insulin sensitivity (3 trials, *n* = 86): NS	Very low certainty * Evidence rated down for inconsistency (1 level), imprecision (1 level), and indirectness (1 level).
**CHROMIUM**					
Zhao, F., et al. 2021 [40] (MA) Inception–July 2020	N = 509 (10 trials) P = RCTs in people with T2D with lab values FBG ≥ 140 mg/dL, HbA1c ≥ 6.9%, triglyceride ≥ 125 mg/dL; I = chromium supplementation; C = placebo; O = HbA1c, FBG, triglycerides	5 different forms of chromium supplements: Cr- containing milk powder, Cr-enriched yeast, chromium nicotinate, brewer’s yeast and chromium picolinate. Doses of chromium ranged from 42 to 1000 μg per day. Duration of intervention ranged from 90 days to 25 weeks. (*Placebo*)	FBG: NS (10 trials, *n* = 522) ↓HbA1c: MD −0.54% (CI −0.98 to −0.09, 9 trials, *n* = 481)		**Very low certainty** Quality of the evidence was downgraded one level for “risk of bias” due to high risk of bias for blinding and unclear allocation concealment; two levels for “inconsistency” due to varying point estimates, inconsistent direction of effect, limited overlap of confidence intervals and point estimates, and high heterogenetiy; one level for “imprecision” due to small sample sizes, and one level for “publication bias”
**PROBIOTICS**					
Cao, D., et al. 2021 [38] (MA) From inception–May 2020	N = 1948 (31 trials, 17 in people with T2D) P = RCTs in people with prediabetes, T2D or GDM; I = probiotics or synbiotics; C = placebo; O = FBG, HbA1c, fasting insulin, HOMA-IR, HOMA-B, and QUICKI Search: May 2020	Single-strain formulation was used in 4 studies and bacteria from Lactobacillus (including Lactobacillus sporogenes) and Bifidobacterium genera were included in probiotic formulations in all 17 and 9 of the included studies, respectively. 7 studies—synbiotics 10 studies—probiotics Dose range: 1 × 10^8^ CFU to 1.00001 × 10^12^ CFU per day. Duration: 6 weeks to 6 months (*Placebo*)	↓FBG: WMD −9.48 mg/dL (CI −16.24 to −2.72, *n* of trials NR, *n* = 1016) ↓HbA1c: WMD −0.43% (CI −0.69 to −0.18, *n* of trials NR, *n* = 635)		**Not provided** by review authors, and GRADE assessment not possible due to insufficient reporting of individual RCT risk of bias for subgroups with T2D. Most (>50%) included trials for all disease groups were at unclear or high risk of bias, publication bias could not be ruled out, and heterogeneity was low.
**ZINC**					
Wang, X., et al. 2019 [42] (MA) From inception–February 2019	N-1700 (32 trials, 19 in people with T2D) P = RCTs in people with T2D, GDM, obesity, prediabetes; I = Zinc supplementation; C = placebo or co-supplementation only; O = FBG, 2-h postprandial glucose (2h-PG), fasting insulin, HOMA-IR, HbA1c, or hs-CRP.	Zinc sulphate, gluconate, amino chelate, oxide, and acetate; in some cases, the anion was not specified. Dose range: 4–240 mg/d; median: 30 mg/d); mean 35 mg/d. Duration: 1 to 12 months. (*Placebo or co-supplement only*)	↓FBG: WMD −20.34 mg/dL (CI −29.04 to −11.64, 12 trials, *n* = 752) HbA1c: NS (8 trials, *n* = 639) PPG: NS (5 trials, *n* = 256)	HOMA-IR (4 trials, *n* = 234), fasting insulin (5 trials, *n* = 292): NS	**Very low certainty** Quality of the evidence was downgraded one level for “risk of bias” due to limited appropriate sequence generation, blinding, and reporting of withdrawals and dropouts; two levels for “inconsistency” due to high heterogeneity, no consistent direction of effect, and varying point estimates; one level for “indirectness” due to insufficient timeframe in some trials; and one level for “imprecision” due to small sample sizes
**MAGNESIUM**					
Verma, H. and Garg, R. 2017 [39] (MA) Inception–June 2016	N = 1694 (28 trials; 17 T2D) P = RCTs in people with T2D/high risk of T2D; I = Magnesium (organic or inorganic) for at least 1 month; C = placebo; O = T2D associated CVD risk factors (FBG, FPI, HbA1C, TC, HDL, LDL, TG, SBP and DBP)	Form of magnesium supplementation included Mg pidolate, citrate, aspartate, chloride, lactate, sulphate and oxide. Dosage ranged from 31.5 mg to 1006 mg of elemental Mg. Duration of intervention ranged from 4 to 24 weeks (*Placebo*)	↓FBG: WMD −6.253 mg/dL (CI −10.602 to −1.904, 15 trials, *n* = 773) HbA1c: NS (7 trials, *n* = 505)	Fasting insulin: NS	**Not provided** by review authors, and GRADE assessment not possible due to insufficient reporting of individual RCT risk of bias for subgroups with T2D. All studies were at unclear risk of bias for allocation concealment, and none were at high risk of bias for sequence generation or selective reporting. Risk of bias was generally low for blinding of participants, personnel and outcome assesors. Heterogeneity was moderate, and there was no evidence of publication bias.
**POLYPHENOLS**					
Jeyaraman, M., et al. 2020 [32] (MA) For Cochrane database: Inception –December 2018 Other databases: Inception–April 2018	N = 50 (3 trials) P = RCTs in people with T2D (for mixed studies, at least 80% of participants had to be adults with T2D); I = oral resveratrol (any regimen); C = placebo, anti-diabetic medications (OHA, insulin, herbal or nutrient supplements), diet/exercise or no treatment; O = adverse events (primary); diabetes-related or all-cause mortality, diabetes complications, HbA1c, FBG, insulin sensitivity, etc. (secondary).	Resveratrol Dose range: 10 mg, 150 mg, or 1000 mg daily. Duration: 4 to 5 weeks. (*Placebo*)	FBG: NS (2 trials, *n* = 33) HbA1c: NS (2 trials, *n* = 33)	HOMA-IR: NS (2 trials, *n* = 36)	Very low certainty * Downgraded by one level because of indirectness (surrogate outcome and insufficient time frame) and by two levels because of serious imprecision (low median sample size and small number of studies, CI ranging between benefit and harm)
Raimundo, A., et al. 2020 [33] (MA) Initial search: Inception–November 2016, and updated in Jane 2018	N = 1200 (20 total,14 in people with T2D) P = RCTs in people with prediabetes or T2D; I = pure (poly)phenol or an enriched fraction of (poly)phenols (4 weeks or more for glucose, 12 weeks or more for HbA1c); C = placebo; O = FBG, HbA1c, insulin, HOMA-IR, IAPP/amylin, glucagon, and C-peptide. Included cross-over trials.	3 trials: Polyphenol mixture (from passion fruit, grape, pine tree park, among others- doses of 125–2093 mg/day) 5 trials: Resveratrol (doses of 40–1000 mg/day) 3 trials: Isoflavones (doses of 33–100 mg/day) 2 trials: Flavanols (doses of 560–1270 mg/day) 1 trial: Anthocyanin (392 mg/day) Duration: 4 to 52 weeks. (*Placebo*)	↓FBG: MD −5.86 mg/dL (CI −11.34 to −0.39, 13 trials, *n* = 740) Subgroup analysis: ↓FBG in those taking anti-diabetic medication: MD − 10.17 (CI −17.59 to −3.75, 6 trials, *n* = 378) No subgroup analysis conducted in people with T2D for HbA1c	Insulin (9 trials, *n* = 552) and HOMA-IR (7 trials, *n* = 489): NS	**Not provided** by review authors, and GRADE assessment not possible due to insufficient reporting of individual RCT risk of bias for subgroups with T2D. 12/14 trials were at moderate-high risk of bias. Heterogeneity was moderate and there was no evidence of publication bias for the included trials for all disease groups.
**Ω-3 PUFAs**					
Gao, C., et al. 2020 [35] (MA) Inception–May 2019	N = 820 (12 trials) P = RCTs in people with T2D; I = Fish oil supplementation alone; C = placebo; O = TG, TC, HDL-C, LDL-C, FBG, FPI, HbA1c, and HOMA-IR.	n3-PUFAs in fish oil Dose range n-3 PUFA: 0.3 g/d to 10.08 g/d. Duration: 3 weeks to 6 months. (*Placebo*)	FBG: NS at any time point; ≤1 month (3 trials, *n* = 102); 1–3 months (6 trials, *n* = 451) or >3 months (4 trials, *n* = 381) HbA1c: NS at any time-point; ≤1 month (1 trial, *n* = 20), 1–3 months (5 trials, *n* = 329), or >3 months (4 trials, *n* = 309)	HOMA-IR: NS at any time-point; ≤1 month (2 trials, *n* = 82), 1–3 months (2 trials, *n* = 224) or >3 months (4 trials, *n* = 381) Fasting insulin: NS at any time-point; ≤1 month (2 trials, *n* = 61), 1–3 months (2 trials, *n* = 224), or >3 months (4 trials, *n* = 381)	**Very low certainty** Quality of the evidence was downgraded one level for “risk of bias” due to unclear risk of bias across random sequence generation, allocation concealment, and blinding; one level for “inconsistency” due to limited overlap of confidence intervals and point estimates and inconsistent direction of the effect; one level for “imprecision” due to small sample sizes; and one level for “publication bias”
O’Mahoney, L., et al. 2018 [36] (MA and MR) Inception–July 2017	N = 1187 T2D (45 total, 31 with glycaemic outcomes) P = Parallel or cross-over RCTs in people with T2D; I = n3-PUFAs including in diet as long as dosage and duration could be determined; C = placebo; FBG, HbA1c, fasting insulin, HOMA-IR and C-peptide, lipid profile, inflammatory markers, BP.	Ω-3 PUFAs in capsule/liquid or diet (sardine-enriched) form. Origin of n3-PUFAs not reported. All trials used EPA, DHA, or a combination. Dose range: 0.40 to 18.00 g. Duration: 2 to 104 weeks (14/33 RCTs were >3 months duration) (*Placebo*)	FBG: NS (28 trials, *n* = 1702) ↓HbA1c: Effect size −0.27 (CI −0.48 to −0.06, 31 trials, *n* = 2021) In “leave one out” sensitivity analysis for HbA1c, removal of two RCTs attenuated the statistical significance of the results to non-significant.	HOMA-IR, fasting insulin: NS	**Very low certainty** Quality of the evidence was downgraded one level for “inconsistency” due to high heterogeneity, inconsistent direction of effect and limited overlap of confidence intervals and point estimates; one level due to insufficient timeframe in some trials; and one level for “imprecision” due to small sample sizes and confidence interval not consistent with benefit
Ebada, M., et al. 2019 [34] (MA) Inception–May 2017, and updated on April 2018	N = 553 (10 trials, 8 T2D) P = RCTs in people with T1D or T2D; I = 1) RCTs with DM patients (both T1 and T2); alpha-lipoic acid (ALA); C = placebo; HbA1c, FBG, PPG, HDL, LDL, TG, TC, HOMA, Glutathione peroxidase, and waist circumference.	Alpha-lipoic acid oral or intravenous. Dose range: 300–600 mg/d. The follow-up duration ranged from three weeks to six months. Duration of intervention NR. (*Placebo*)	FBG: NS (6 trials, *n* = 322), HbA1c: NS (6 trials, *n* = 316) PPG: NS (3 trials, *n* = 190):		**Very low certainty** Quality of the evidence was downgraded by one level for “inconsistency” due to limited overlap of confidence intervals and point estimates, moderate heterogeneity and inconsistent direction of effect; one level for “indirectness” due to limited applicability and insufficient timeframe for some trials; one level for “imprecision” due to small sample sizes; and one level for “publication bias”
**VITAMIN D**					
Hu, Z., et al. 2019 [37] (MA) From inception of database–March 2018	N = 747 (19 trials) P = RCTs in people with T2D; I = Vitamin D; C = placebo; O = FBG, insulin, HbA1c, HOMA-IR.	Vitamin D (type NR) Dose range: 1000 IU/d to 300,000 IU single IM injection Duration: 4 wk to 12 mo. Duration <6 mo considered short-term. (*Placebo*)	FBG: NS (14 trials, *n* = 289), HbA1c: NS (19 trials, *n* = 747) Short-term (<6 mo): ↓ HbA1c: SMD −0.17% (CI −0.27 to −0.04, 15 trials, *n* = 1059) Long term: NS.	↓ HOMA-IR: SMD −0.60 (CI −0.79 to −0.42, 9 trials, *n* = 425) ↓Fasting insulin: SMD −0.49 (CI −0.68 to −0.31, 9 trials, *n* = 436) Short-term (<6 mo): ↓ HOMA-IR: SMD −0.75 (CI −0.97 to −0.53, 8 trials, *n* = 405) ↓ Insulin: SMD −0.57 (CI −0.78 to −0.35, 8 trials, *n* = 389). Long term: NS.	**Very low certainty** Quality of the evidence was downgraded one level for “risk of bias” because allocation concealment was unclear; two levels for “inconsistency” because the direction of the effect was inconsistent and there was high heterogeneity; and one level for “imprecision” due to small sample sizes
**FOLATE**					
Lind, M., et al. 2019 [41] (MA) Pubmed from 1953–March 2018 Web of Science from 1900–March 2018 EMBASE from 1974–March 2018.	N = 572 (29 total, 8 T2D) P = Parallel and cross-over RCTs with no restriction on health condition; folate supplementation; C = placebo; O = glucose, insulin, HOMA-IR, or HbA1c.	Folate given as adjuvant therapy (alongside antidiabetic medication ± insulin). Two studies combined folate with B12 and B6. Dose range: 0.25 mg folate and 5 mg folic acid/d, with most studies using dosage of 5 mg. Duration: 2 weeks to 2 years, with the majority of studies lasting between 4 and 8 weeks. (*Placebo*)	FBG: NS (6 trials, *n* = 309) HbA1c: NS (7 trials, *n* = 482). No differences found on subgroup analysis by baseline folate concentration, however this was conducted on the pooled sample which included people without T2D.	HOMA-IR: NS (9 trials, *n* = 431)	**Very low certainty** Quality of the evidence was downgraded two levels for “risk of bias” due to unclear allocation concealment and blinding of outcome assessment, and high risk of selective reporting; one level for “inconsistency” due to varying point estimates and limited overlap of confidence intervals and point estimates; one level for “indirectness” due to insufficient timeframe and inclusion of co-interventions in some trials; and one level for “imprecision” due to small sample sizes.

Legend-Review Type: MA: Meta-analysis; MR: Meta-regression; UR: Umbrella review; SR: Systematic Review. Interventions: DHA: docosahexaenoic acid; EPA: eicosapentaenoic acid. Outcome Measures: FBG: fasting blood glucose; PPG: post-prandial glucose; HbA1c: glycosylated haemoglobin; HOMA-IR: Homeostatic Model Assessment for Insulin Resistance; FPI: fasting plasma insulin; QUICKI: Quantitative Insulin-Sensitivity Check Index; HDL-C: high density lipoprotein cholesterol; LDL-C: low density lipoprotein cholesterol; NR = not reported; NS = results not statistically significant; OHA: oral hypoglycaemic agents; TG: triglycerides; TC: total cholesterol; SBP: systolic blood pressure; DBP: diastolic blood pressure. * GRADE assessment conducted by systematic review authors; ↓=decrease in outcome measure when intervention was compared to control.

**Table 2:** AMSTAR 2 rating of included reviews.

Nutrient Supplement	Reference	Critical Flaws ^a^	AMSTAR 2 Rating ^b^
Vitamin C	Mason, et al. 2021 [43]	7	Low
Chromium	Zhao, et al. 2021 [40]	2, 7, 15	Critically low
Probiotics	Cao, et al. 2021 [38]	2, 7, 13	Critically low
Zinc	Wang, et al. 2019 [42]	7, 9, 11	Critically low
Magnesium	Verma, H., Garg, R. [39]	2	Low
Polyphenols	Jeyaraman, et al. 2020 [32]	None	High
	Raimundo, et al. 2020 [33]	7	Low
PUFAs	Gao, et al. 2020 [35]	7, 11, 13	Critically low
	O’Mahoney, et al. 2018 [36]	7	Low
	Ebada, et al. 2019 [34]	7	Low
Vitamin D	Hu, et al. 2019 [37]	4, 7, 9, 11, 13	Critically low
Folate	Lind, et al. 2019 [41]	7	Low

^a^ Refers to the item numbers of the AMSTAR 2 critical domains that the review has failed to satisfy. ^b^ Refers to the AMSTAR 2 rating of overall confidence in the results of the review: High, Moderate, Low, Critically low.

## Data Availability

Data is available from the research team upon reasonable request.

## Supplementary Materials

The following are available online at https://www.mdpi.com/article/10.3390/nu14112295/s1, Table S1: Search strategy used based on the PICO framework.
