# Supplementary material for: Efficacy and Safety of Nutrient Supplements for Glycaemic Control and Insulin Resistance in Type 2 Diabetes: An Umbrella Review and Hierarchical Evidence Synthesis
Source: Nutrients. 2022 May 30;14(11):2295. doi: 10.3390/nu14112295 (PMC9182772; doi:10.3390/nu14112295)
Supplement: Supplementary file 1 [file nutrients-14-02295-s001.zip › nutrients-1733734-supplementary.pdf]

Supplementary

Table S1. Search strategy used based on the PICO framework.

**Population**<br>Diabetes mellitus OR diabetes* OR NIDDM OR T2D* or (non next insulin* next depend) OR (noninsulin* next depend) OR (non next insulindepend*)<br><br>**Intervention**<br>vitamin* OR mineral* OR nutrient* OR food supplement* OR meal replacement* OR nutritional supplement* OR health supplement* OR multivitamin* OR omega 3 OR fish oil* OR alpha lipoic acid OR alpha linolenic acid OR alpha linoleic acid OR eicosapentaenoic OR docosahexaenoic OR fatty acid* OR amino acid* OR taurine OR s-adenosyl methionine OR creatine OR acetylcysteine OR cysteine OR probiotic* OR tryptophan OR tocopherol OR alphatocopherol OR carotene OR retinol OR thiamine OR riboflavin OR niacin OR niacinamide OR nicotinic acid OR pantothenic OR pyridox* OR biotin OR methylfolate OR 5-MTH* OR levomefolic acid OR folate OR folinic acid OR folic acid OR inositol OR cyanocobalamin OR methylcobalamin OR cobalamin OR ascorbic acid OR cholecalciferol OR iron OR ferrous OR tocopherols OR trace element OR calcium OR phosphorus OR magnesium OR potassium OR manganese OR zinc OR selenium OR boron OR chromium OR lycopene OR isoflav* OR flavonoid* OR bioflavonoid* OR micronutrient OR carnitine<br>**Comparator**<br>random* OR placebo OR control* or adjunc* or clinical trial*<br>**Outcomes**<br>glycaemic control* OR blood glucose* OR HbA1c* OR HOMA-IR* OR plasma insulin OR lipoprotein cholesterol* OR triglycerides* OR total cholesterol* OR blood pressure*
